# Tertiary Care Center Trends in Colonic Stent Placement over the Past Decade

**DOI:** 10.3390/cancers16193309

**Published:** 2024-09-27

**Authors:** Wassem Y. Juakiem, Kelita Singh, Andrew Ofosu, Daryl Ramai, Alana Persaud, James H. Tabibian, Eduardo Rodrigues-Pinto, Mohit Girotra, Monique T. Barakat

**Affiliations:** 1 Division of Gastroenterology, Stanford University, Stanford, CA 94305, USA; wassem.y.juakiem.mil@health.mil (W.Y.J.); singhke@upstate.edu (K.S.);; 2 Division of Gastroenterology, Hepatology and Endoscopy, Brigham and Women’s Hospital, Boston, MA 02115, USA; 3 Division of Gastroenterology and Hepatology, SUNY Downstate, Brooklyn, NY 11203, USA; ayp152@jefferson.edu; 4 Adventist Health Glendale, Glendale, CA 91206, USA; 5 David Geffen School of Medicine at UCLA, Los Angeles, CA 90095, USA; 6 Division of Gastroenterology, Centro Hospitalar São João, Porto, Portugal; edu.gil.pinto@gmail.com; 7 Digestive Health Institute, Swedish Medical Center, Seattle, WA 98104, USA; 8 Division of Pediatric Gastroenterology, Lucille Packard Children’s Hospital, Stanford University Medical Center, Palo Alto, CA 94305, USA; 9 Medicine and Pediatrics, Divisions of Adult/Pediatric Gastroenterology & Hepatology, Stanford University Medical Center, 300 Pasteur Drive, Stanford, CA 94305, USA

**Keywords:** colonic stent, malignant obstruction, benign obstruction

## Abstract

**Simple Summary:**

Endoscopic stent placement within the colon is a common procedure but is understudied. Here, we studied all of the colon stents placed over the past 10 years at our institution and found a trend toward more colon stents being placed in the last 5 years of the study period and colon stents being placed in younger patients in the last 5 years of the study period. We also show that colon stents can be safely and effectively placed for benign reasons (not cancer).

**Abstract:**

Introduction: Colonic endoluminal stent placement is a commonly utilized and effective endoscopic approach for the management of malignant large bowel obstruction and is an emerging approach for the management of some benign etiologies of large bowel obstruction. However, recent studies evaluating the evolution of clinical scenarios and patient populations for which stenting is performed in real-world practice are lacking. Methods: We assessed colonic stent utilization patterns in a tertiary care academic medical center over the past 10 years. We analyzed the demographics and patient and procedure characteristics of the initial (first half of study period) and latter (second half of the study period) procedures to assess trends over time using standard descriptive statistics. Results: Our analysis was notable due to its provision of some novel insights. The frequency of colonic stent placement procedures increased significantly over time by comparison of the procedure volume for the initial 5-year interval (22 colonic stent procedures) relative to the latter 5-year interval (49 colonic stent procedures) (*p* = 0.03). The median age of patients who underwent colonic stent placement was significantly lower in the latter 5 years, compared with the initial 5 years of the study period (mean of 81.41 vs. 58.73 years, respectively, *p* < 0.001). The increased diversity of indications for colonic stent placement was also noted over time. Conclusions: Our data highlight the evolution of colonic stent placement in tertiary care practice over time and are notable for some interesting trends, including the increased utilization of colonic stent placement over time, the broadening of indications for colonic stent placement to include benign indications, and lower patient age at the time of colonic stent placement over time. These findings will help inform the clinical practice of colonic stent placement and provide a foundation to guide future research on the topic.

## 1. Introduction

Colonic endoluminal stent placement is a commonly utilized and effective endoscopic approach for the management of malignant large bowel obstruction [1]. In the United States, where colorectal carcinoma is common, the incidence of large bowel obstruction in patients with colon cancer ranges up to 29% [2,3]. In prior decades, malignant large bowel obstruction was managed by emergent surgical intervention (e.g., colostomy) to relieve the obstruction; however, this approach is associated with higher peri-operative mortality and morbidity rates relative to elective surgical management (following bowel preparation to clear stool burden), independent of tumor stage and patient age [4,5].

In addition to providing decompression of the large bowel, thus obviating the need for emergency surgery, colonic stent placement allows improvement in the patient’s clinical condition, adequate oncological staging, good colonic preparation, performance of an elective surgery by an experienced surgical team, the possibility of a laparoscopic approach, and shorter latency to the initiation of chemotherapy. Disadvantages of colonic stent placement include the need for the technical expertise of an advanced endoscopist, which may not be available at all centers; the need for fluoroscopy guidance; and possible worse oncologic outcomes. Furthermore, some angulated and proximal colonic locations may be less amenable to colonic stent placement. Since their introduction in the 1990s, colonic stents have been refined to include self-expanding elements and to enhance the ease of endoscopic deployment. Trials have been conducted to evaluate the role of these colonic self-expanding metallic stents (SEMSs) as a bridge to surgical intervention for malignant large bowel obstruction, and findings have supported the superiority of endoscopic stent placement as an initial intervention relative to direct emergent surgical intervention [4,5]. Most of the literature concerns left-sided obstructing colon cancer, excluding (distal) rectal cancers; however, SEMSs may also be successfully placed in malignant obstructions of the proximal/right colon [6].

There is also an emerging role of colonic endoluminal stent placement in benign large bowel obstruction [7,8,9]. Indeed, SEMS placement has been reported for the management of large bowel obstruction due to diverticulitis, inflammatory bowel disease, anastomotic colonic strictures and endometriosis. Although this emerging role for endoluminal stent placement has not yet been rigorously studied, it has been increasingly reported in case reports and case series [7,9,10,11,12]; however, it should only be considered on a case-by-case basis after multidisciplinary discussion.

While studies have found that colonic stent placement is safe and effective for the management of large bowel obstruction in malignant large bowel obstruction, recent studies evaluating the evolution of clinical scenarios and patient populations for which stenting is performed in real-world practice are lacking. In the present study, we aim to evaluate colonic stent utilization patterns in a tertiary care academic medical center over the past 10 years to characterize current practice and guide future research, in order to assess the evolution of clinical scenarios and patient populations for which stenting is performed in real-world practice.

## 2. Methods

### 2.1. Study Design

All colonoscopy and flexible sigmoidoscopy procedures with endoluminal stent placement performed from 2010 to 2020 at our tertiary care academic medical center were included in this analysis. Data were retrospectively extracted from a prospectively maintained endoscopy database and the STAnford Research Repository (STARR). This work was approved by the Stanford University Medical Center Institutional Review Board (IRB # 57500). The endoscopy database and search tool were used to identify all patients who underwent endoscopic colonic stent placement and to identify the characteristics of these patients and stent placement procedures. Because procedure-level data were not fully available using the STARR database search system, the endoscopy database was used to review and analyze details of specific endoscopic procedures for colonic stent placement. A limited subset of patients underwent placement of a second colonic stent several months after the first stent, typically in the setting of palliative stent placement with subsequent tumor/tissue ingrowth in the months following stent placement leading to stent occlusion; these second procedures were excluded from the analysis. In summary, inclusion criteria were an endoscopic procedure during which colonic stent placement was performed. The exclusion criterion was a history of being a colonic stent patient prior to the procedure performed at our institution. All patients who underwent endoscopic colonic stent placement and were identified through the STARR database search were included in this analysis, with the exception of those meeting the single exclusion criterion delineated above.

### 2.2. Technical Success, Clinical Outcomes and Adverse Event Assessment

The goal of this study was to identify and analyze all colonic stents placed during the study period (2010–2020) and to analyze the initial (first half of study period) and latter (second half of the study period) procedures to assess trends over time.

Technical success was assessed by the procedure description in the procedure note and was classified as successful or unsuccessful based on whether the endoluminal stent was placed in the intended location, traversing the lesion/obstruction.

Clinical outcome was assessed by the evaluation of post-procedure inpatient and outpatient notes in the medical records for indications of symptom improvement consistent with the relief of bowel obstruction post-procedure.

The occurrence of any adverse event was documented, and adverse events were planned to be classified by type (e.g., oozing/bleeding, perforation).

### 2.3. Technique

Colonoscopy and flexible sigmoidoscopy with stent placement were performed using standard techniques. All procedures during the study period were performed with anesthesia support (monitored anesthesia care or general anesthesia). Procedures were performed by a therapeutic endoscopist, typically with an advanced endoscopy fellow participating in the procedures.

The colonic stent placement procedure is notable due to the following aspects: Prior to colonic stent placement procedures, all patients undergo cross-sectional imaging for assessment of the site and characteristics of colonic obstruction. In preparation for the procedure, for patients who have a bowel obstruction, oral bowel preparation is avoided and, instead, serial tap water enemas are administered to the patient to clear stool burden distal to the site of colonic obstruction. Our typical recommendation is at least 4 enemas, with the number and volume of enemas to be determined by the output associated with the enemas. For example, if the output is clear/liquid, then additional enemas may not be required, but if the output contains solid or semi-solid stool, then additional enemas may be necessary. Pre-anesthesia assessment is also conducted prior to colonoscopy or flexible sigmoidoscopy for colonic stent placement.

The colonic stent placement procedure is typically performed with the patient in the left lateral decubitus or supine position on the fluoroscopy table. The colonoscope or therapeutic gastroscope is then advanced through the colon to the site of obstruction using the water immersion technique with only minimal carbon dioxide when necessary to avoid over-distension of the colon proximal to the stricture. Once the site of obstruction is visualized, a long 0.035″ guidewire is advanced through the narrowed lumen of the colon. The location of the guidewire is evaluated on fluoroscopy images and a cannula is then advanced beyond the area of obstruction. Contrast is injected to opacify the colon proximal to the stricture, the stricture itself and the colon distal to the stricture. Once the length and characteristics of the stricture are determined, the self-expanding metallic stent length is determined. The length is selected to allow for at least 2 cm of stent proximal to and distal to the stricture. Self-expanding metallic stents exert additional radial force following stent placement, which can help facilitate patency, but this radial force is also associated with some foreshortening of the stent. For strictures at flexures (e.g., hepatic flexure or splenic flexure), longer stents may be necessary to ensure that the full length of the stricture is traversed and to allow for foreshortening of the stent during stent expansion. At the time of stent deployment, the flexible stent deployment sheath, which is a flexible catheter that houses the SEMS, is advanced over the guidewire (the guidewire that traverses the stricture). This stent over the guidewire is advanced through the scope. Deployment is then accomplished under endoscopic and fluoroscopic guidance, with the goal of having at least 2 cm of stent proximal and 2 cm of stent distal to the stricture. Upon full deployment, fluoroscopy should demonstrate a ‘waist’ (narrowing) of the stent at the site of the stricture. Colonic stent placement often occurs on an inpatient basis, and patients often remain admitted for at least a day following colonic stent placement to evaluate for the resolution of obstruction symptoms and to monitor any acute adverse events associated with colonic stent placement. At the time of discharge from the hospital, patients are advised to eat a low-residue diet with the avoidance of insoluble fiber and to take laxatives (often Miralax) to facilitate liquid/semi-liquid stool consistency (and thus avoid solid stool occluding the stent lumen).

### 2.4. Statistical Analysis

All analyses were conducted using the SAS Enterprise online platform (SAS Institute Inc., Cary, NC, USA) and Microsoft Excel (Version 16.89).

Continuous data were analyzed for normality using the Kolmogorov–Smirnov test. For normally distributed data (e.g., age, gender), mean and standard deviation (SD) are reported. Student’s T-test and chi-squared test were performed to assess statistical significance. All reported *p*-values are 2-sided, and all comparisons attained statistical significance at *p* < 0.05.

## 3. Results

### 3.1. Patient Demographics

Over the study period, 72 patients underwent endoscopic placement of a colonic stent at our tertiary care academic medical center. Patient characteristics are summarized in Table 1. Patients ranged from 31 to 99 years of age, with a median age of 66.1 years (SD 20.1 years). In total, 52.7% (38/72) of patients were female, and this gender distribution did not significantly change over the study period. Patient ethnicity was notable, with 59.7% (43/72) Caucasian patients, 18% (13/72) Asian patients and 12.5% (9/72) Black patients, and the remainder of patients identified with another ethnicity or preferred not to state. Patient ethnicity did not significantly change over the study period. The insurance/payer status of patients who underwent colonic stent placement was similarly stable over the study period.

### 3.2. Trends in Colonic Stent Procedure Volume and Characteristics

The frequency of colonic stent placement procedures increased significantly over time, when analyzed by stratified annual procedure volume and by comparison of the procedure volume for the initial 5-year interval (23 colonic stent procedures) relative to the latter 5-year interval (49 colonic stent procedures) (*p* = 0.03). The vast majority of colonic stents (90.3%, 65/72) were placed to address large bowel obstruction in the left colon, and this preferentially left-sided location of colonic stent placement did not significantly differ in the initial vs. latter study period. The remainder of stents (9.7%, 7/72) were placed in the transverse colon and hepatic flexure region.

### 3.3. Trends in Patient Age

The median age of patients who underwent colonic stent placement was significantly lower in the latter 5 years, compared with the initial 5 years of the study period (mean of 81.41 vs. 58.73 years, respectively, *p* < 0.001). Notably, the youngest patient to undergo colonic stent placement in the initial 5 years was 61, and the youngest patient to undergo colonic stent placement in the latter 5 years was 31.

### 3.4. Trends in Procedure Indication

The increased diversity of indications for colonic stent placement was noted over time. Colonic stent placement procedures were exclusively performed for malignant indications during the first half of the study period (23 colonic stent procedures). During the last half of the study period, 16.3% (8/49) of colonic stent placement procedures were performed for benign indications, as described below.

### 3.5. Technical Success and Adverse Events 

The vast majority of procedures were technically successful (94.4%). The technical success of procedures remained high throughout the study period and did not significantly differ between the early and late study periods (*p* = 0.814). All patients in whom a stent was placed had evidence of clinical improvement (relief of obstruction) within 3 days following stent placement. No procedure-associated adverse events were identified for patients/procedures included in this analysis.

### 3.6. Procedures for Malignant Indication 

**Figure 1:**
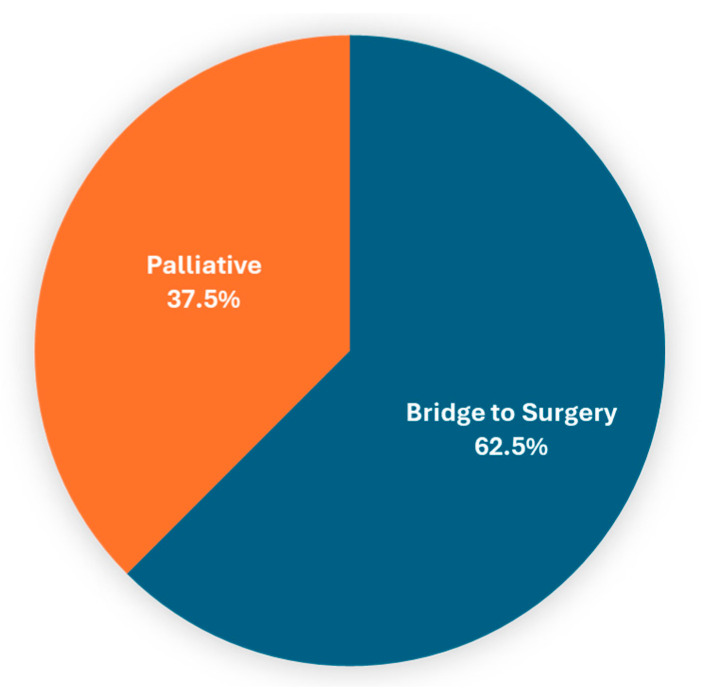
Clinical goal of colonic stent placement procedures performed for malignant indication.

The analysis of patients with malignant indication for colonic stent placement (n = 64) was notable for younger patients in the most recent 5-year interval. In total, 93.8% (60/64) were technically successful, with 62.5% (40/62) performed as a bridge to surgical intervention and 37.5% (22/62) performed for the management of obstruction (Figure 1).

### 3.7. Procedures for Benign Indication

**Figure 2:**
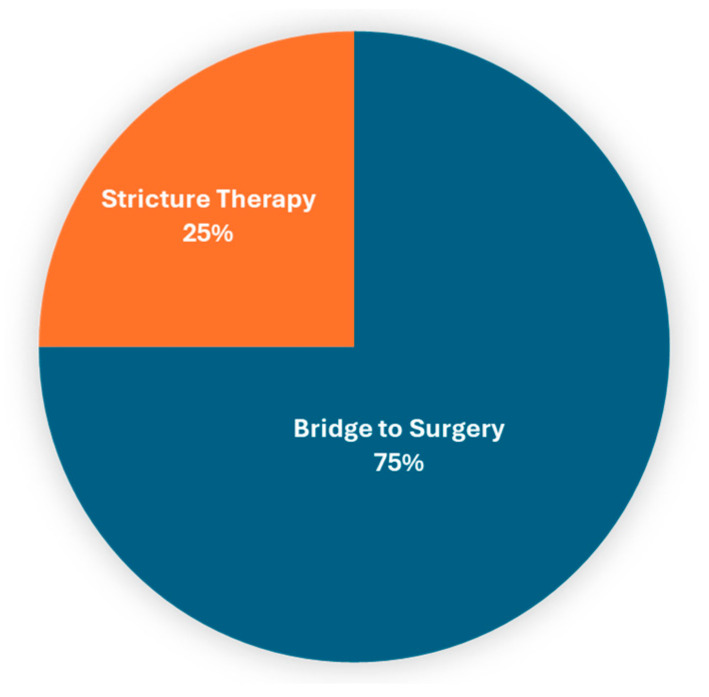
Clinical goal of colonic stent placement procedures performed for benign indications.

The analysis of patients with benign indications for colonic stent placement (n = 8) was notable for a range of indications, including large bowel obstruction due to diverticulitis, inflammatory bowel disease, anastomotic colonic strictures and endometriosis. Technical success was 100% and 7/8 (87.5%) of patients experienced clinical improvement in their symptoms following colonic stent placement. Of these procedures, 75% (6/8) were performed as a bridge to surgical intervention, and 25% (2/8) were placed with a definitive intent for stricture therapy (Figure 2). Symptom improvement was reported in the medical record for all patients who underwent colonic stent placement for benign indications, and those who underwent colonic stent placement as a bridge to surgical intervention eventually underwent surgical resection.

## 4. Discussion

The endoscopic placement of colonic stents is considered safe and effective for the management of large bowel obstruction and has been best studied in the setting of malignant obstruction [1,3]. Colonic stent placement has been integrated into clinical practice since it was first described in the 1990s, traditionally as a bridge to the surgical resection of a malignant lesion or for palliation in patients whose colorectal malignancy was not amenable to surgical resection [2]. Since the integration of colonic stent placement into mainstream endoscopic practice, studies and meta-analyses have attested to the safety of this practice and characterized patient symptom trajectory and outcomes following colonic stent placement; however, the evolution of practice patterns surrounding colonic stent placement over time have not been well characterized [4,5]. In the present study, we analyze colonic stent utilization patterns in a tertiary care academic medical center over the past decade to highlight the evolution of the practice and guide future research. Our main findings include (i) an escalating frequency of colonic stent placement during the study period, (ii) a broadening of the range of indications for colonic stent placement and (iii) a significant decline in patient age at the time of colonic stent placement.

The trends indicating an escalating frequency of colonic stent placement and expansion of the range of indications for which colonic stents were placed over the study period are of interest. This evolution of colonic stent placement is consistent with that of other endoscopic devices and interventions. Often, after an initial period of successful use, the device becomes more commonly used and the range of conditions for which the device is utilized expands. The use of colonic stents for benign indications remains controversial and relatively high risk based on available data [7,9,10,11,12]; however, our single-center findings support the potential for colonic stent placement for benign indications to be safely integrated into endoscopic practice. We found uniform technical success in colonic stent placement procedures performed for benign indications of large bowel obstruction due to diverticulitis, inflammatory bowel disease, anastomotic colonic strictures and endometriosis. Aside from the 25% of colonic stent procedures performed for anastomotic colonic strictures, which were performed for therapeutic dilation of the stricture, all of these colonic stents placed for benign indications were placed as a bridge to surgical intervention. It is worth noting that colonic stent placement was performed by experienced endoscopists at a site with a robust gastroenterological oncology and surgical oncology program during both the initial and subsequent 5-year study period; thus, we do not anticipate that any substantial learning curve effect was present or influenced the data.

We further identified a trend of decreasing patient age at the time of colonic stent placement over the study period. This trend was notable for all-comers and for the subset of patients who underwent colonic stent placement for malignant bowel obstruction. This finding is novel and aligns with the literature demonstrating that, since the 1990s, the rate of colorectal cancer has more than doubled among adults younger than 50 [13,14,15,16]. The reasons underlying this trend are unclear and represent an area of active research. It is notable that younger patients with colorectal cancer appear more likely to present with advanced-stage disease, perhaps because they do not undergo usual surveillance [13,14,15,16]. These young patients who present with advanced-stage disease may be more likely to require colonic stent placement as a bridge to surgical resection following neoadjuvant chemotherapy and/or require palliative stent placement for obstructive symptoms.

Limitations of this study include its single-center and retrospective nature, rendering it susceptible to sampling bias and reliant upon the accuracy of medical record and procedure report documentation, and meaning that it has a potential lack of generalizability to other institutions, as well as other limitations associated with retrospective studies. Due to limitations in the medical records, we were unable to definitively ascertain the type of colonic stent placed for each patient and we could not accurately identify all patients for whom our advanced endoscopy service was consulted for consideration for colonic stent placement. Our data are thus limited to those procedures which were ultimately performed for colonic stent placement. Due to limitations in medical records and procedure coding, it is further possible that a subset of procedures which were attempted but not completed (e.g., due to inadequate bowel preparation) were not captured. It would be of interest to study this in a prospective manner to avoid these limitations, and to study other practice settings and multiple centers to understand whether these findings are generalizable to other centers and practice settings. Of note, some emerging machine learning-based approaches for image-level tasks may be relevant to colonic stent placement, stricture identification and colonoscopy, but these approaches were not directly used or evaluated in the present study [17,18,19,20,21,22,23,24].

Here, we analyzed colonic stent utilization patterns in a tertiary care academic medical center over the past decade. In conclusion, these data highlight the evolution of the intervention and are notable due to some interesting trends, including the broadening of indications for colonic stent placement to include benign indications and lower patient ages at the time of colonic stent placement in the most recent 5 years, relative to the first 5 years of the study period, consistent with the trend of colorectal cancer incidence increasing in younger patients. These findings will help inform the clinical practice of colonic stent placement and provide a foundation to guide future research on the topic.

## Figures and Tables

**Table 1:** Patient and procedure characteristics.

Median Age (in years)	66.1 (range 31–99)
Gender (# (%))	38/72 (52.7%)
Ethnicity	Caucasian: 43/72 (59.7%)
	Asian: 13/72 (18%)
	Black: 9/72 (12.5%)
	Other/Not Stated: 7/72 (9.7%)
Indication for Colonic Stent Placement	Malignant Obstruction: 64/72 (88.9%)
	Benign Obstruction: 8/72 (11.1%)
Colonic Stent Placement Volume	Number of Procedures in Initial 5 Years of Study Period: 23
	Number of Procedures in Last 5 Years of Study Period: 49

## Data Availability

The original contributions presented in the study are included in the article; further inquiries can be directed to the corresponding author.
